# Dietary Polyphenols in Relation to Gut Microbiota Composition in Saudi Arabian Females

**DOI:** 10.3390/metabo13010006

**Published:** 2022-12-20

**Authors:** Munirah N. Alsuhaibani, Ghadeer S. Aljuraiban, Esra’a A. Aljazairy, Manal Abudawood, Syed D. Hussain, Abdullah Alnaami, Shaun Sabico, Nasser M. Al-Daghri, Sara Al-Musharaf

**Affiliations:** 1 Department of Community Health Sciences, College of Applied Medical Sciences, King Saud University, Riyadh 11451, Saudi Arabia; 2 Department of Clinical Laboratory Sciences, College of Applied Medical Sciences, King Saud University, Riyadh 11433, Saudi Arabia; 3 Chair for Biomarkers of Chronic Diseases, Riyadh Biochemistry Department, College of Science, King Saud University, Riyadh 11451, Saudi Arabia

**Keywords:** polyphenols, gut microbiota, body weight, obesity, BMI, Bacteroidetes, Firmicutes

## Abstract

Polyphenols may modulate gut microbiota; however, limited studies have examined this relationship relative to obesity. We aim to investigate the association between polyphenol intake and gut microbiota composition in relation to obesity indices among Saudi Arabian females. This study included 92 adults stratified by body mass index (BMI) into controls (BMI ≥ 18.5–24.9 kg/m^2^; *n* = 48) and cases (BMI ≥ 30.0 kg/m^2^; *n* = 44), and further divided into high and low polyphenol intake by median intake (252 mg/1000 kcal/day). Fecal samples were collected to analyze the gut microbiota composition via the whole-genome shotgun sequencing technique. Results showed that *Flavonifractor plautii* and *Clostridium bolteae* were positively correlated with polyphenol intake in the total sample (*r* = 0.22, *p* = 0.03; *r* = 0.28, *p* = 0.01, respectively). There were inverse correlations between *Blautia wexlerae* and polyphenol intake (*r* = −0.56, *p* < 0.01) in the case group, and between *Bacteroides thetaiotaomicron* and polyphenol intake (*r* = −0.45, *p* = 0.03) in the control group. Those in the case group with low polyphenol intake, and those with high waist-to-hip ratio (WHR; ≥0.83), showed significantly lower alpha-diversity than those in the control group with normal WHR (<0.83), (*p* < 0.05). Findings suggest that polyphenols are correlated with specific bacteria and may play an important role in the modulation of gut microbiota and obesity management.

## 1. Introduction

Worldwide obesity has nearly tripled since the 1970s [1]. In the Eastern Mediterranean region, overweight and obesity rates have increased dramatically, especially among women, reaching 50% and 24%, respectively, in 2016 [2,3]. In Saudi Arabia, the prevalence of obesity among the adult population was 20% in 2019, with a higher rate in women compared to men [4]. The increasing rate of obesity, especially among women of reproductive age, is worrying, since a mother’s nutritional status was linked to the risk of chronic diseases in newborns [5,6]. Obesity generally develops due to multiple factors, such as a sedentary lifestyle, genetic predisposition, and unhealthy dietary patterns [7], with recent findings suggesting that other factors, such as micronutrient deficiencies and alterations in gut microbiota [8,9], may contribute to obesity.

The gut microbiota are microorganisms inhabiting the human gastrointestinal tract [10]. The composition and diversity of gut microbiota are crucial aspects, since the imbalance of gut microbiota has been involved in the development of various health conditions, such as obesity, diabetes mellitus, and cardiovascular diseases [11]. One of the main contributors to microbiota imbalance is dietary intake, which may impact the microbiota diversity and composition [10]. The quantity and the type of consumed macronutrients and some micronutrients, such as polyphenols, can affect the gut microbiota composition in response to the metabolites of the dietary components [12].

Polyphenols are a large group of secondary metabolites found in fruits, vegetables, coffee, tea, and many other foods [13], that possess various properties such as antioxidant, anti-inflammatory, anti-obesogenic, and antilipidemic effects [13,14]. More specifically, preclinical studies have shown that intake of polyphenols, specifically catechins, anthocyanins, and proanthocyanidins, may modify the gut microbiota community through exerting prebiotic-like effects [15]. It is proposed that ingested polyphenols play a role in the gut microbiota by stimulating the growth of bacteria recognized as prebiotic targets [15] and hindering the growth of pathogenic ones [16]. Likewise, gut microbiota may act on polyphenols and generate a large array of metabolites [17]. Consequently, the interaction between polyphenols and the microbiota-generated metabolites may exert positive effects on human health, such as attenuating the development of obesity via altering the gut microbial composition [17].

Currently, and to the best of our knowledge, only four human clinical trials have investigated the effects of polyphenols on gut microbiota and obesity [18,19,20,21], with limited available studies on habitual polyphenol intake. Further understanding of this association is important for the management of obesity and preservation of gut health; therefore, the aim of this study was to investigate the association between dietary polyphenols and gut microbiota composition relative to obesity indices in Saudi Arabian women, using the whole genome shotgun sequencing (WGS) to assess gut microbial profiles.

## 2. Materials and Methods

### 2.1. Study Participants

The current study is part of a previously published case–control study that identified the composition of the gut microbiota in relation to obesity [22]. Here, we focused on nutrients related to gut composition, specifically, dietary polyphenols. We included Saudi females randomly selected from King Saud University (KSU) between January 2019 and March 2020. Participants were recruited from the female campus of KSU through flyers distributed across colleges, word of mouth, faculty members’ assistance, and social media. We included females aged 18 years or older and excluded participants with characteristics that may confound results, e.g., pregnancy, diagnosis with blood, psychological, endocrine, oncological, or gastrointestinal diseases (e.g., acute, or chronic diarrhea), and those who consumed multivitamin or vitamin B12, and used antibiotics in the last six months before fecal sample collection. Based on these criteria, a total of (*n* = 308) were excluded (Figure S1), bringing participants to a total of 92, divided into a control group (Body Mass Index (BMI) ≥ 18.5 to 24.9 kg/m^2^; *n* = 48) and a case group (BMI ≥ 30.0 kg/m^2^; *n* = 44). Ethical approval was granted by the Institutional Review Board (IRB) of King Khaled University Hospital, Riyadh (IRB number: E-019-3625). All participants provided written informed consent, with the right to withdraw from the study at any time. After consenting, participants were given fecal sample containers and appointments were scheduled at the clinic to provide anthropometric measurements, dietary data, and fecal samples.

### 2.2. Sample Size Calculation

The sample size was computed at a 5% significance level and 80% power to evaluate the composition of gut microbiota and associated differences among Saudi college students as previously described [23]. To achieve Firmicutes/Bacteroidetes ratios of (0.9 ± 0.4) and (1.7 ± 1.7) in the control and case groups, respectively, the sample size needed for each group was 46 participants. The required total sample size was 99 participants, including a 10% dropout rate, comparable to previous studies [24,25].

### 2.3. Anthropometric Measurements

Body weight, height, waist, and hip circumferences were measured. The bioelectrical impedance was used to analyze body composition (770 Bioelectrical Impedance Analyzer: In-Body, Seoul, South Korea). Weight was recorded to the nearest 0.1 kg using an international standard scale, with participants barefoot and wearing light clothes (Digital Pearson Scale; ADAM Equipment Inc., Oxford, CT, USA). The height was measured to the nearest 0.5 cm with the same scale (Digital Pearson Scale), while participants were facing the scale and standing barefoot. Waist circumference was measured using the midway between the lowest rib and the umbilicus, while hip circumference was measured at the great trochanter, with the legs close together, using non-stretchable tape. The nearest 0.5 cm of these measurements was reported. Waist-to-hip ratio (WHR) was calculated by dividing the two values. Participants with WHR < 0.83 were classified as “normal WHR”, and those with WHR ≥0.83 were classified as “high WHR” [26]. All measurements were recorded twice and the average reading was used.

### 2.4. Gut Microbiota Composition

#### 2.4.1. DNA Extraction

Fresh fecal samples were collected and stored instantly at −80 °C. The DNA was extracted from 0.25 gm of frozen fecal aliquots following the QIAamp PowerFecal DNA Isolation Kit protocol (Qiagen, Hilden, Germany). For higher yields, DNA was eluted in 100 mL of the C6 buffer, and a nanodrop spectrophotometer (NanoDrop Technologies, Wilmington, NC, USA) was used to determine purity (260/280 ratio) and concentration (≥1.6). The volume of extracted DNA samples was (≥12.5 μL), stored at −20 °C for further analyses.

#### 2.4.2. Library Preparation and Sequencing

DNA libraries were built using the Nextera XT DNA Library Preparation Kit (Illumina, San Diego, CA, USA) and Nextera Index Kit (Illumina), with a total DNA input of 1 ng. A proportionate amount of Illumina Nextera XT fragmentation enzyme was used to fragment genomic DNA. Each sample was given a combination of dual indexes, followed by 12 cycles of polymerase chain reaction (PCR) to create libraries. DNA libraries were purified with AMpure magnetic Beads (Beckman Coulter) and eluted in QIAGEN EB buffer. For the quantitative evaluation, a Qubit^®®^ fluorimeter (Thermo Fisher, Milan, Italy) was used. Following the library preparation, samples were sequenced on an Illumina HiSeq 4000 (2 × 150 bp).

#### 2.4.3. Characterization of Microbial Composition

The determination of gut microbiota composition at phylum levels was done by identifying the total bacterial DNA, Bacteroidetes, and Firmicutes DNA, using the WGS [27]. Employing the CosmosID bioinformatics platform (CosmosID Inc., Rockville, MD, USA), unassembled sequencing reads were directly analyzed for multi-kingdom microbiome analysis, profiling of antibiotic resistance and virulence genes, and quantification of the relative abundance of organisms. This system uses curated genome databases and a high-performance data-mining algorithm that rapidly deciphers hundreds of millions of metagenomic sequence reads into the detached microorganisms generating particular sequences. Likewise, the resistome and virulome communities, which represent the array of antibiotic resistance and virulence-associated genes, respectively, in the microbiome, were also determined via querying the unassembled sequence reads against the curated CosmosID antibiotic resistance and virulence-associated gene databases. Alpha-diversity (α-diversity) and beta-diversity (β-diversity) are higher-level measures used to describe the microbiome in a given sample [28]. Alpha-diversity is used to measure microbiome diversity within a single sample to describe the richness and evenness, whereas beta-diversity measures similarity or dissimilarity between communities [28].

### 2.5. Dietary Intake

Trained dietitians used the Saudi food frequency questionnaire (FFQ) during interviews with participants to collect data on habitual food and beverage intake during the past year [29]. Food models and standard household-measures were used to assist participants in estimating their consumed dietary quantities. Food and beverage intake were estimated using an FFQ spreadsheet that was developed for the Saudi populations and included 133 items [29]. To increase the accuracy of dietary estimation, 20% of the study population were asked to fill out 24-h dietary recalls of two non-consecutive days (one weekday and one weekend day) and the multi-pass technique was employed to improve accuracy. Results of Pearson Correlation showed correlation coefficients between macronutrients assessed from the FFQ and the 24-h data, ranging between *r* = 0.42 and *r* = 0.63, which were considered acceptable [30].

### 2.6. Assessment of Polyphenol Intake

The Phenol-Explorer database (www.phenol-explorer.eu) (accessed on 1 January 2020) was used to obtain data on the polyphenol content of foods and beverages [31]. All foods and beverages that contained no, or only trace amounts, of polyphenols, such as food of animal origin, were excluded. For mixed foods that contain polyphenols, the content of polyphenols was calculated based on the ingredients. Retention and yield factors presented in the database were applied to convert polyphenols content in raw food into content in processed food. However, the majority of processed food in the FFQ did not have correspondent retention/yield factors in the Phenol-Explorer database; therefore, the content of polyphenols in raw food were used. The data used to calculate the total polyphenol content corresponded to the high-performance liquid chromatography method [31]. If there were various types of the same food item in the database, and if these were not specified in the FFQ, the most common type consumed by the Saudi population was used.

### 2.7. Statistical Analysis

All data were analyzed using IBM SPSS statistics (version 24; IBM software, Armonk, NY, USA). Quantitative data were checked for normality distribution and skewness. Results were expressed as (mean ± SD) for continuous variables, while frequencies and percentages were reported for categorical variables. Medians and interquartile ranges were used for non-normal variables. Independent sample *t*-test was used to assess the differences between continuous variables. Dietary polyphenol intake was calculated in energy-adjusted terms (i.e., mg of polyphenols per 1000 kcal/day of total energy intake). Polyphenol intake was categorized into low and high intake using the median intake as the cut-off point (≤252 vs. >252 mg/1000 kcal/day). We further divided participants into categories based on polyphenol intake: the case group (low intake: ≤236 vs. high intake: >236 mg/1000 kcal/day); and the control group (low intake: ≤281 vs. high intake: >281 mg/1000 kcal/day) groups. Age-adjusted Pearson’s correlation coefficients between polyphenol intake and gut microbiota were applied. The α-diversity and β-diversity principal-coordinate analyses of shotgun metagenomic sequencing were applied using the online CosmosID bioinformatics program (Rockville, MD). The α-diversity (within-subject diversity) was assessed using the Shannon test to obtain the differences in the relative abundances or richness of the gut bacterial taxa [28]. The β-diversity (between-subject diversity) was assessed using the Bray–Curtis test to explore the variability in community composition [28]. A *p*-value < 0.05 and a confidence interval of 95% was considered statistically significant.

## 3. Results

Ninety-two female participants aged (21.1 ± 1.5) years old have completed the study (Table S1).

### 3.1. Dietary Polyphenols Intake

The median intake of polyphenols was 953 mg/day (252 mg/1000 kcal/day) for the total study participants (Table S1). Polyphenol intake of the case group was lower than the control group but was not statistically significant (236.2 vs. 280.5 mg/1000 kcal/day, respectively; *p* = 0.106). Furthermore, non-statistically significant differences were found between macronutrients, micronutrients, or food groups except for fruits intake, which was higher among the control group compared to the case group (Table S1).

### 3.2. Anthropometrics Measurements

The muscle mass percentage was significantly higher in the control group with higher polyphenols (33 ± 7 mg/1000 kcal/day, *p* = 0.03), compared to the group with low polyphenols (27 ± 10 mg/1000 kcal/day) (Table 1). No statistically significant differences were found for the other anthropometric measurements between the study groups (Table 1).

### 3.3. Gut Microbiota Composition

There were no statistically significant differences in Firmicutes and Bacteroidetes phyla between study groups (Table 1). In the case group, the abundance of *Clostridium bolteae*, *Clostridioides difficile*, and *Bifidobacterium pseudocatenulatum* were significantly lower (*p* = 0.03; *p* = 0.04; *p* = 0.04, respectively) in the high compared to the low polyphenol intake group. Likewise, in the control group, the abundances of Verrucomicrobia, *Akkermansia muciniphila*, and *Bacteroides faecichinchillae* were significantly lower (*p* = 0.03; *p* = 0.02; *p* = 0.20, respectively) in the high compared to low polyphenol intake group (Table 1).

### 3.4. Correlations between Gut Microbiota, Dietary Polyphenol Intake, and BMI

In the total sample, *Flavonifractor plautii* and *Clostridium bolteae* were positively correlated with dietary polyphenol intake (*r* = 0.22, *p* = 0.03; *r* = 0.28, *p* < 0.01, respectively) (Table 2). In the case group with low polyphenol intake, a moderate inverse correlation was observed between polyphenol intake and *Blautia wexlerae* (*r* = −0.56, *p* < 0.01) (Table 3). In the control group with high polyphenol intake, *Clostridium bolteae* were moderately positively correlated with polyphenol intake (*r* = 0.54, *p* < 0.01), while *Bacteroides thetaiotaomicron* was inversely correlated with polyphenol intake (*r* = −0.45, *p* = 0.03) in the low polyphenol intake group.

### 3.5. Gut Microbiota Diversity Analyses

**Figure 1:**
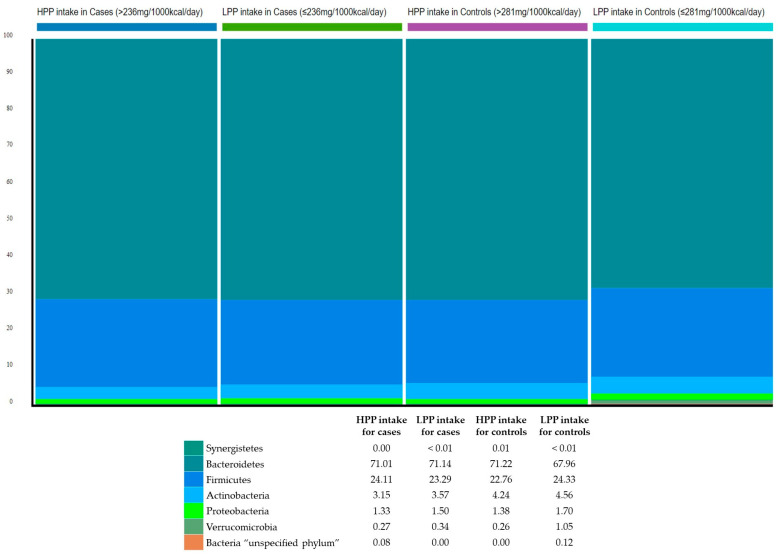
Phylum-level classification of gut microbiota according to categories of polyphenol intake and relative to BMI. HPP, high polyphenols; LPP, low polyphenols.

The relative abundance of the gut microbiota at the phylum level was estimated to explore the bacterial community composition according to polyphenol intake with respect to BMI. The three most abundant bacterial phyla in the study sample were: Bacteroidetes (70.3%); Firmicutes (23.6%); and Actinobacteria (3.9%) (Figure 1). 

**Figure 2:**
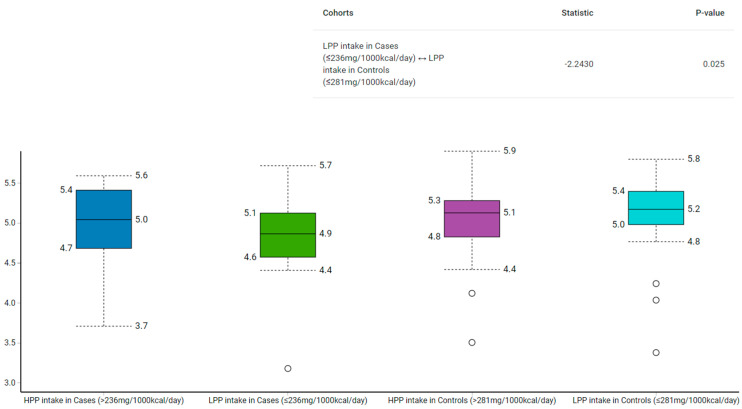
Comparison of gut microbiota α-diversity according to categories of polyphenol intake and relative to BMI using Shannon–Wiener Index. HPP, high polyphenols; LPP, low polyphenols.

**Figure 3:**
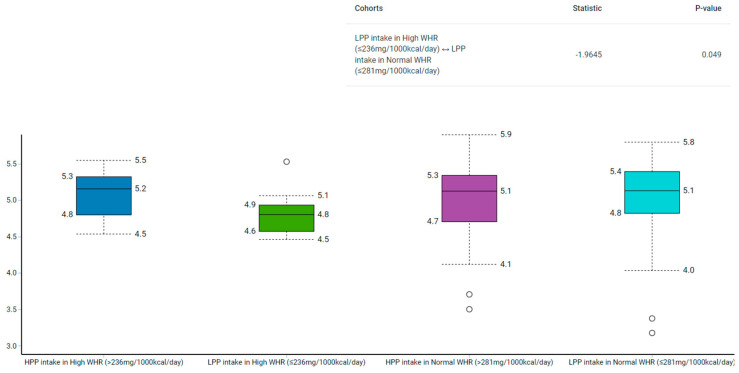
Comparison of gut microbiota α-diversity according to categories of polyphenol intake and relative to WHR using Shannon–Wiener Index. HPP, high polyphenols; LPP, low polyphenols.

Among those with low polyphenol intake, the Shannon–Wiener index revealed that the control group had a higher α-diversity than the case group (*p* = 0.02) (Figure 2). Similarly, the α-diversity was higher in the normal compared to the high WHR group with low polyphenol intake (*p* = 0.05) (Figure 3).

## 4. Discussion

To the best of our knowledge, this is the first study to investigate dietary polyphenol intake in relation to gut microbiota composition relative to obesity indices in a Saudi sample population, with further identification of the α- and β-diversity of the gut microbiota. Dietary polyphenol intake was estimated at 252 mg/1000 kcal/day, higher in the control group compared to the case group. With regards to gut microbiota, we found that *Flavonifractor plautii* and *Clostridium bolteae* were positively and significantly correlated with dietary polyphenol intake in the total sample. In the case group with low polyphenol intake, *Blautia wexlerae* showed a moderate inverse correlation with polyphenol intake, while in the control group, *Bacteroides thetaiotaomicron* exhibited a moderate inverse relationship with polyphenol intake, while *Clostridium bolteae* showed a positive correlation with polyphenol intake. Furthermore, α-diversity of the gut microbiota was lower in both the case group and the group with high WHR, indicating lower gut health [32]. The bacterial diversity, as measured by β-diversity [28], was similar among the study groups.

In the current study, significant correlations between dietary polyphenols and gut microbiota relative to BMI were observed at the species level. However, there were non-significant phylum-level trends within the control group with high polyphenol intake; the Firmicutes phyla was inversely correlated with polyphenol intake (*r* = −0.37, *p* = 0.09), whereas Bacteroidetes phyla revealed a favorable correlation with polyphenol intake (*r* = 0.37, *p* = 0.08). These trends are interesting as Firmicutes bacteria showed adverse effects on health, while Bacteroidetes bacteria revealed a favorable influence on health [33,34]. This provides insight and suggests that a high intake of dietary polyphenols may increase Bacteroidetes and decrease Firmicutes, thus improving gut health.

At the species level, the current study revealed that *Blautia wexlerae* were significantly inversely correlated with polyphenol intake in the case group with low polyphenol intake, whereas in the control group with high polyphenol intake, *Clostridium bolteae* were significantly and positively correlated with polyphenol intake. *Bacteroides thetaiotaomicron* showed significant inverse correlations with polyphenol intake in the low polyphenol intake group. Human studies relating the above-mentioned bacterial species with polyphenol intake and obesity markers are limited [20,35]. Results of our study suggest that the abundance of *Blautia wexlerae* in participants with obesity may be low if polyphenol intake continues to be low. The *Blautia genus* (from Firmicutes phylum) has been shown to alleviate inflammatory as well as metabolic diseases [36]. Depletion of *Blautia wexlerae* in the gut may develop in participants with obesity, contributing to metabolic inflammation and insulin resistance [37]. This may explain the low levels of *Blautia wexlerae* in the case group, which is further exacerbated by lower polyphenol intake. Human studies on *Blautia wexlerae* and its association with polyphenols are scarce. A 4-week crossover trial reported an increase in the abundance of *Blautia wexlerae* in the prediabetes group following supplementation with polyphenols [38]. However, the supplements contained both polyphenols and prebiotics, which may have confounded the results [38].

In the control group with low polyphenol intake, *Bacteroides thetaiotaomicron* showed significant inverse association with polyphenol intake. This finding suggests that if polyphenol intake remains low, *Bacteroides thetaiotaomicron* abundance may also be low. To our knowledge, no previous studies have reported the relation between *Bacteroides thetaiotaomicron* and polyphenol intake. *Bacteroides thetaiotaomicron* is one of the most prominent bacteria in the human digestive system [39], and aids in shaping the nutritive environment of the gut microbiota by degrading complex dietary fiber polysaccharides and producing short-chain fatty acids [40]. The reduction in the abundance of *Bacteroides thetaiotaomicron* and other *Bacteroides* species has been linked to gut inflammation [41]. Hence, our findings highlight the role of polyphenols in relation to beneficial bacteria such as *Bacteroides thetaiotaomicron*, which in turn enhances gut health.

Contrary to expectations, the current study reported a significant positive relationship between *Clostridium bolteae* and polyphenol intake in the control group with high polyphenol intake. When the natural intestinal barrier is compromised, *Clostridium bolteae*, a part of the human intestinal microbiota, can cause intra-abdominal infections [42]. A previous RCT found a significant reduction in *Clostridium* species (−2.3 ± 0.79, *p* < 0.05) in Spanish adults with metabolic syndrome after red wine consumption, compared to baseline results [20]. A systematic meta-analysis review reported that tea and apple intake were the most effective dietary polyphenol sources in decreasing the abundance of *Clostridium* pathogen species as well as intake of fruits and vegetables [35]. However, this contradictory finding may be attributed to the overall low dietary polyphenol intake in the current study. The median daily polyphenol intake of the study participants was 953 mg/day (252 mg/1000 kcal/day). As per our knowledge, in the Middle Eastern region, no previous investigations have estimated the dietary polyphenol intake except for one study in Iran [43]. Participants of the current study had lower intakes of dietary polyphenols compared to an Iranian sample (252 vs. 346 mg/1000 kcal/day, respectively) [43]. Internationally, a systematic review estimated the overall dietary polyphenol intake of 45 studies from different regions, mainly Europe, to about 360 mg/1000 kcal/day, higher than the current study [44]. It is worth noting that there is currently no national or international recommended daily intake of dietary polyphenols [45,46]. Furthermore, the differences in gut microbiota composition observed between studies may be attributed to different lifestyle factors and the used DNA extraction protocol. A recent study by He et al. analyzed the gut microbial profile of 7009 participants (homogeneous for ethnicity) of the Chinese province of Guangdong [47] and revealed that the gut microbiota composition and its relationship with metabolic disease was strongly dependent on the geographical region, noting that associations found in a district could not be extrapolated to other districts [47]. Costea et al. [48] have examined 21 DNA extraction protocols on the same fecal samples to explore the extent to which these different procedures would influence the quantification of microbial community composition. They reported that the variations in DNA extraction protocol have the largest impact on the observed microbial composition [48].

The α-diversity used to measure the richness and evenness of gut microbiota [28], has been linked to various acute and chronic diseases [49,50]. We found that participants in the case group with low polyphenol intake had lower α-diversity than their counterparts from the control group (median: 4.9 vs. 5.2; *p* = 0.03). To illustrate, the median polyphenol intake was higher in the control group with low polyphenols (281 mg/1000 kcal/day) compared to their counterparts from the case group (236 mg/1000 kcal/day). Similarly, those with low polyphenol intake in the high WHR group had lower α-diversity than those with low polyphenol intake in the normal WHR group (median: 4.8 vs. 5.1; *p* = 0.05). However, as both groups had low polyphenols, but the control group had more polyphenols than the case group, α-diversity analysis indicate the likelihood that polyphenol intake may play a role in the modulation of gut bacteria.

The β-diversity, used to assess variation in comparison to bacterial communities, did not show significant differences between polyphenol intake and obesity indices including BMI and WHR. However, this is not particularly surprising if we consider the high inter-variation in the gut microbiota between the participants. Previous research has suggested that low α-diversity is a reliable indication of disease-associated dysbiosis [51,52,53]. Furthermore, Claesson et al. reported that better health status is probably linked with higher α-diversity [54]. It is worthy to note that we are not aware of other studies that assessed α-diversity of gut microbiota with polyphenol intake.

The mechanism by which dietary polyphenols play a role in lowering obesity markers may be attributed to the modulation of gut microbiota. However, the precise mechanism has yet to be elucidated. A growing body of evidence suggests that dietary polyphenols may induce white adipose tissue browning and thermogenesis [55], mediated by gut microbiota [56]. Gut microbiota may modulate thermogenesis through metabolizing the bile acid by converting primary bile acids into secondary bile acids [17]. The latter could act as a signal to molecules to activate certain receptors, increasing the browning white adipose tissue and energy expenditure [17]. Therefore, the interaction between dietary polyphenols, gut microbiota, and thermogenesis may be used to develop a new strategy for managing obesity.

The current study has several strengths. We investigated the association between dietary polyphenols and gut microbiota relative to body weight using WGS for taxonomic identification to species level, which is considered to be a gold standard technique when compared to other methods [57]. Furthermore, extensive measures were applied to increase the validity and reliability of collected data such as in-person interviews and repeated anthropometrics. However, a number of potential limitations should be considered. This observational study cannot establish causality; thus, more experimental research is needed to confirm a cause-and-effect relationship. Furthermore, the findings cannot be generalized to the Saudi population as the study included young females only. The stratification of participants may have led to loss of statistical power. Additionally, the reliance on subjective data, (i.e., FFQ and 24-h) instead of biochemical measures, may not have reflected the actual dietary intake. However, previous studies that combined both FFQ and 24-h, as in our case, showed better accuracy of the estimates of dietary intake [58].

In conclusion, this study suggests that dietary polyphenol intake may play a considerable role in modulating the gut microbiota of Saudi Arabian females. However, the beneficial effects of polyphenols on human health remain inconclusive. Future RCTs are needed to explore the underlying mechanisms behind the association between dietary polyphenols, the specific gut bacteria identified in this study, and obesity indices.

## Figures and Tables

**Table 1:** Anthropometric measurements and gut microbiota of participants stratified by dietary polyphenol intake and BMI, *n* = 92.

Variables	Cases			Controls		
	Low Polyphenols (≤236 mg/1000 kcal/Day)	High Polyphenols (>236 mg/1000 kcal/Day)	*p*-Value	Low Polyphenols (≤281 mg/1000 kcal/Day)	High Polyphenols (>281 mg/1000 kcal/Day)	*p*-Value
Anthropometric Measurements						
BMI (kg/m^2^)	36.1 ± 4.4	36.0 ± 5.0	0.10	22.0 ± 2.0	21.4 ± 1.7	0.30
WHR (ratio)	0.8 ± 0.1	0.7 ± 0.1	0.60	0.7 ± 0.0	0.7 ± 0.1	0.66
Body fat (%)	51.0 ± 3.3	51.1 ± 3.3	0.94	35.1 ± 4.6	34.4 ± 6.3	0.67
Muscle mass (%)	26.6 ± 2.0	26.6 ± 1.9	0.94	26.6 ± 10.4	32.5 ± 7.2	**0.03**
Gut Microbiota						
Firmicutes	0.21 (0.15–0.32)	0.23 (0.16–0.30)	0.98	0.21 (0.17–0.32)	0.20 (0.17–0.27)	0.46
*Blautia wexlerae*	0.01 (0.0038–0.01)	0.01 (0.0034–0.01)	0.30	0.0045 (0.0025–0.01)	0.01 (0.0038–0.01)	0.29
*Flavonifractor plautii*	0.0008 (0.0003–0.0018)	0.0007 (0.0004–0.0011)	0.49	0.0005 (0.0003–0.0008)	0.0008 (0.0004–0.0018)	0.07
*Clostridium bolteae*	0.0004 (0.0002–0.0013)	0.0000 (0.0000–0.0005)	**0.03**	0.0003 (0.0000–0.0006)	0.0005 (0.0001–0.0007)	0.29
*Faecalibacterium prausnitzii*	0.02 (0.02–0.03)	0.02 (0.01–0.02)	0.08	0.026 (0.01–0.03)	0.02 (0.01–0.02)	0.16
*Clostridioides difficile* ^§^	0.01 ± 0.05	0.0001 ± 0.0001	**0.04**	0.0001 ± 0.0001	0.01 ± 0.04	0.14
Bacteroidetes	0.74 (0.63–0.83)	0.72 (0.66–0.81)	0.98	0.72 (0.55–0.77)	0.74 (0.66–0.79)	0.35
*Bacteroides faecichinchillae* ^§^	0.004 ± 0.02	0.0001 ± 0.0001	0.97	0.01 ± 0.03	0.0001 ± 0.0001	**0.02**
*Bacteroides thetaiotaomicron*	0.01 (0.0023–0.01)	0.01 (0.0035–0.01)	0.73	0.01 (0.0038–0.01)	0.01 (0.0033–0.01)	0.61
Actinobacteria	0.02 (0.02–0.05)	0.02 (0.01–0.04)	0.50	0.03 (0.02–0.06)	0.04 (0.01–0.07)	0.85
*Bifidobacterium pseudocatenulatum*	0.0017 (0.0000–0.0057)	0.0001 (0.0000–0.0015)	**0.04**	0.0000 (0.0000–0.0048)	0.0005 (0.0000–0.0031)	0.33
Verrucomicrobia	0.0005 (0.0000–0.0043)	0.0003 (0.0000–0.0015)	0.59	0.0019 (0.0004–0.0128)	0.0003 (0.0000–0.0038)	**0.03**
*Akkermansia muciniphila*	0.0005 (0.0000–0.0043)	0.0003 (0.0000–0.0015)	0.76	0.0029 (0.0004–0.01)	0.0003 (0.0000–0.0038)	**0.02**
Proteobacteria	0.01 (0.01–0.02)	0.01 (0.01–0.02)	0.45	0.01 (0.01–0.02)	0.01 (0.01–0.02)	0.42
Fusobacteria	0.0000 (0.0000–0.0000)	0.0000 (0.0000–0.0000)	0.15	0.0000 (0.0000–0.0000)	0.0000 (0.0000–0.0000)	1.00
F/B (ratio)	0.29 (0.19–0.49)	0.32 (0.20–0.44)	0.96	0.28 (0.22–0.59)	0.28 (0.21–0.41)	0.40

Note: Data presented as mean ± SD for normal variables and median (1st quartile–3rd quartile) for non-normal continuous variables. *p*-value < 0.05 considered significant (bold). ^§^ Mean ± SD are shown for certain significant variables to clarify which group had higher/lower value. Bacteria written in non-italic indicate phylum, while those in italic indicate species. Body mass index (BMI), waist-to-hip ratio (WHR), Firmicutes/Bacteroidetes (F/B).

**Table 2:** Correlations between gut microbiota and polyphenol intake, *n* = 92.

Variables	Polyphenol Intake (mg/1000 kcal/Day)	*p*-Value
Firmicutes	−0.02	0.86
*Blautia wexlerae*	−0.07	0.49
*Flavonifractor plautii*	0.22	**0.03**
*Clostridium bolteae*	0.28	**<0.01**
*Faecalibacterium prausnitzii*	−0.18	0.10
*Clostridioides difficile*	0.01	0.95
Bacteroidetes	0.06	0.59
*Bacteroides faecichinchillae*	−0.10	0.36
*Bacteroides thetaiotaomicron*	−0.08	0.43
Actinobacteria	−0.08	0.46
Verrucomicrobia	−0.11	0.30
*Akkermansia muciniphila*	−0.12	0.26
Proteobacteria	−0.07	0.53
Fusobacteria	−0.10	0.35
F/B (ratio)	0.02	0.83

Note: Data presented as Pearson correlation coefficient adjusted for age. *p* < 0.05 was considered significant (bold). Bacteria written in non-italic indicate phylum while those in italic indicate species. Firmicutes/Bacteroidetes (F/B).

**Table 3:** Correlations between gut microbiota and dietary polyphenol intake across each category.

Variables	Cases				Controls			
	Low Polyphenols (≤236 mg/1000 kcal/Day)		High Polyphenols (>236 mg/1000 kcal/Day)		Low Polyphenols (≤281 mg/1000 kcal/Day)		High Polyphenols (>281 mg/1000 kcal/Day)	
	*r*	*p*-Value	*r*	*p*-Value	*r*	*p*-Value	*r*	*p*-Value
Firmicutes	−0.29	0.20	0.36	0.11	0.04	0.87	−0.37	0.09
*Blautia wexlerae*	−0.56	<0.01	0.15	0.52	0.28	0.20	−0.28	0.20
*Flavonifractor plautii*	−0.04	0.88	0.29	0.20	0.14	0.52	0.15	0.50
*Clostridium bolteae*	−0.16	0.49	0.28	0.22	−0.11	0.60	0.54	**<0.01**
*Faecalibacterium prausnitzii*	0.39	0.08	−0.12	0.60	−0.28	0.19	−0.19	0.39
*Clostridioides difficile*	0.14	0.54	−	−	0.18	0.40	0.00	0.99
Bacteroidetes	0.29	0.20	−0.31	0.16	−0.19	0.38	0.37	0.08
*Bacteroides faecichinchillae*	0.17	0.46	−0.11	0.63	0.26	0.24	−	−
*Bacteroides thetaiotaomicron*	0.19	0.42	−0.29	0.20	−0.45	**0.03**	0.04	0.86
Actinobacteria	−0.11	0.63	−0.02	0.94	0.29	0.18	−0.24	0.27
Verrucomicrobia	0.11	0.63	−0.21	0.36	0.31	0.15	−0.27	0.22
*Akkermansia muciniphila*	0.11	0.63	−0.22	0.35	0.27	0.22	−0.27	0.22
Proteobacteria	−0.17	0.47	−0.04	0.86	0.35	0.10	−0.04	0.87
Fusobacteria	−0.35	0.12	−	−	−	−	−	−
F/B (ratio)	−0.26	0.26	0.36	0.11	0.10	0.66	−0.26	0.23

Note: Data presented as Pearson correlation coefficient adjusted for age. *p* < 0.05 considered significant (bold). Bacteria written in non-italic indicate phylum while those in italic indicate species. Firmicutes/Bacteroidetes (F/B).

## Data Availability

The raw data supporting the conclusion of this article will be made available by the authors, without undue reservation.

## Supplementary Materials

The following supporting information can be downloaded at: https://www.mdpi.com/article/10.3390/metabo13010006/s1, Figure S1: Flow diagram of the study participants; Table S1: General characteristics of the study participants.

## Abbreviations

RCT: Randomized Controlled Trial; WGS: Whole Genome Shotgun; KSU: King Saud University; IRB: Institutional Review Board; WHR: Waist to Hip Ratio; FFQ: Food Frequency Questionnaire; BMI: Body Mass Index.
