# Supplementary material for: Dietary Polyphenols in Relation to Gut Microbiota Composition in Saudi Arabian Females
Source: Metabolites. 2022 Dec 20;13(1):6. doi: 10.3390/metabo13010006 (PMC9864957; doi:10.3390/metabo13010006)
Supplement: Supplementary file 1 [file metabolites-13-00006-s001.zip › metabolites-2100279-supplementary.pdf]

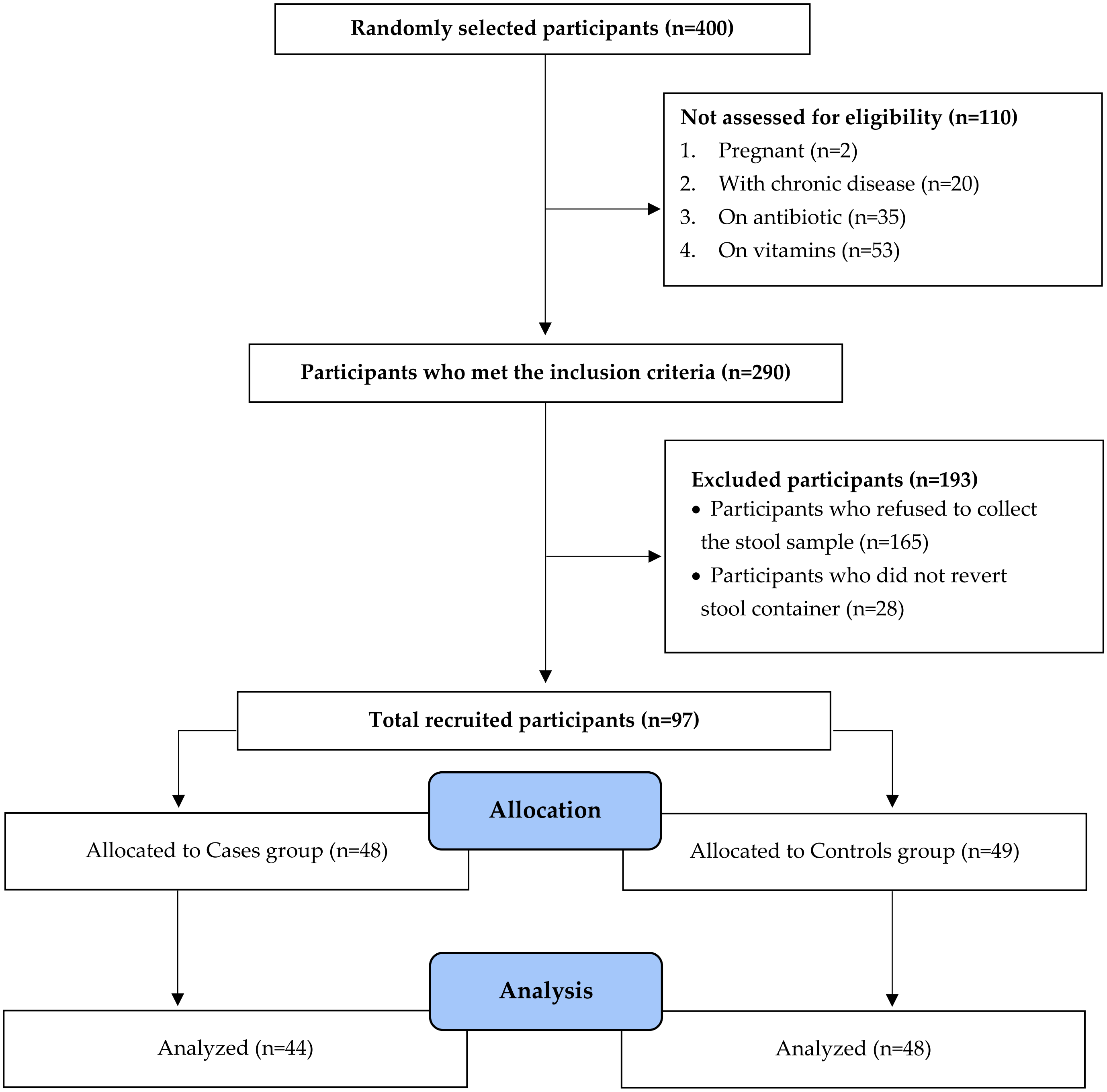

Figure S1. Flow chart of participation in the study.

Table S1. General characteristics of the study participants

Variable	Total (n=92)	Controls (n=48)	Cases (n=44)	*p*-value
Age (y)	21.1 ± 1.5	20.6 ± 1.1	21.6 ± 1.7	**0.002**
Age at menarche (y)	12.4 ± 1.0	12.5 ± 1.0	12.3 ± 1.1	0.312
**Anthropometric Measurements**				
Height (cm)	157.7 ± 5.2	158.0 ± 5.7	157.3 ± 4.5	0.475
Weight (kg)	70.7 ± 19.7	54.3 ± 6.0	89.1 ± 11.8	**<0.001**
BMI (kg/m^2)	28.5 ± 8.0	21.7 ± 1.9	36.0 ± 4.7	**<0.001**
Waist (cm)	80.4 ± 17.4	67.7 ± 4.3	94.5 ± 15.4	**<0.001**
Hip (cm)	109.2 ± 16.4	96.4 ± 7.6	123.5 ± 10.8	**<0.001**
WHR (ratio)	0.73 ± 0.09	0.71 ± 0.06	0.75 ± 0.11	**0.014**
Body Fat (%)	42.5 ± 9.4	34.8 ± 5.5	51.1 ± 3.3	**<0.001**
Protein (kg)	7.6 ± 1.1	6.9 ± .7	8.5 ± .9	**<0.001**
Skeletal muscle mass (kg)	21.0 ± 3.5	18.6 ± 2.2	23.6 ± 2.6	**<0.001**
Muscle mass (%)	28.2 ± 7.0	29.6 ± 9.3	26.6 ± 1.9	**0.041**
Total body water (L)	28.7 ± 4.3	25.8 ± 2.7	31.9 ± 3.3	**<0.001**
Body water (%)	42.1 ± 6.8	47.7 ± 4.0	35.9 ± 2.3	**<0.001**
**Dietary Intake**				
Dairy (gm/1000 kcal)	91.4 (51.8 - 131.5)	84.3 (47.6 - 122.1)	99.1 (52.7 - 141.9)	0.257
Fruit (gm/1000 kcal)	103.2 (61.0 - 194.1)	124.3 (70.5 - 206.7)	84.4 (47.6 - 172.9)	**0.034**
Vegetables (gm/1000 kcal)	182.5 (133.4 - 251.3)	207.6 (143.3 - 279.0)	161.9 (113.2 - 236.7)	0.061
Grains (gm/1000 kcal)	137.1 (96.0 - 179.6)	133.6 (97.6 - 172.9)	144.3 (94.9 - 186.0)	0.574
Polyphenols (mg/day)	952.8 (539.5 - 1404.8)	1063.6 (734.5 - 1421.1)	845.0 (428.8 - 1405.0)	0.135
Polyphenols (mg/1000 kcal/day)	251.5 (188.2 - 358.1)	280.5 (213.9 - 374.4)	236.2 (169.3 - 333.6)	0.106

Note: Data presented as mean ± SD for normal variables, median (1st quartile – 3rd quartile) for non-normal variables. *p*-value <0.05 considered significant. Body mass index (BMI), waist-to-hip ratio (WHR).
